# Supplementary material for: Cysteine availability tunes ubiquitin signaling via inverse stability of LRRC58 E3 ligase and its substrate CDO1
Source: Nat Commun. 2026 May 7;17:4196. doi: 10.1038/s41467-026-72524-3 (PMC13156300; doi:10.1038/s41467-026-72524-3)
Supplement: Supplementary file 1 — Supplementary Information [file 41467_2026_72524_MOESM1_ESM.pdf]

Supplementary Information:

Cysteine availability tunes ubiquitin signaling via inverse stability of LRRC58 E3 ligase and its substrate CDO1

Andree, Stier, et al.

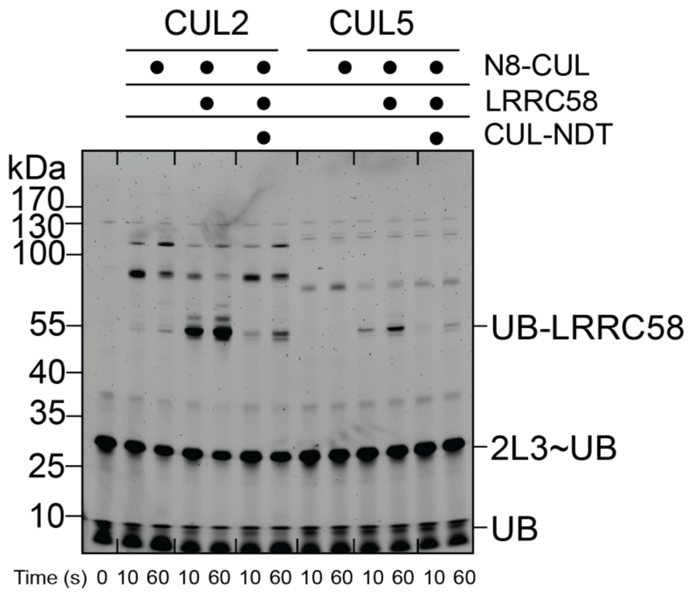

Supplementary Figure 1. LRRC58 is autoubiquitylated in complex with a neddylated-CRL. In vitro reconstitution of LRRC58 autoubiquitylation by neddylated CUL2 and CUL5. LRRC58-EloB/C was also incubated prior to ubiquitylation with non-catalytically active CUL2/5-N-terminal Domain (NTD), which outcompete the active cullin scaffold for LRRC58 binding and thus decrease the observed autoubiquitination. Assay was performed with fluorescent wild-type ubiquitin. Source data provided as Source Data file.

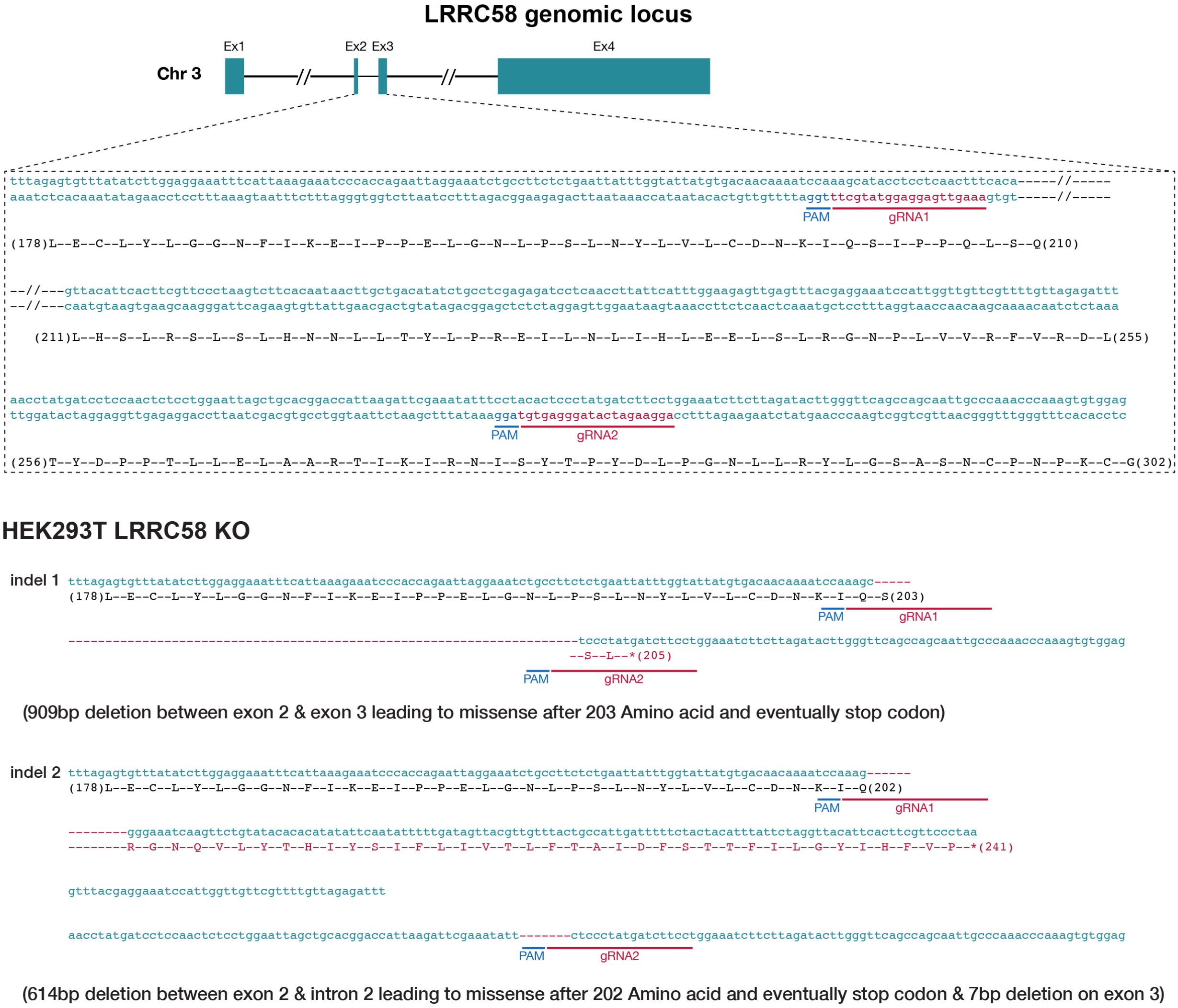

Supplementary Figure 2. CRISPR Cas9 mediated knockout of LRRC58 and schematic representation of its genomic locus for different knockout clones used in the study. Schematic representation of genomic locus of LRRC58 showing exons structure, location of target guide RNAs (gRNAs) pairs (represented in red), and PAM sequence (represented in blue). Insertions/deletions of LRRC58 knockout in HEK293T cells and its potential missense effects on LRRC58 protein expression are shown (missense amino acids shown in red).

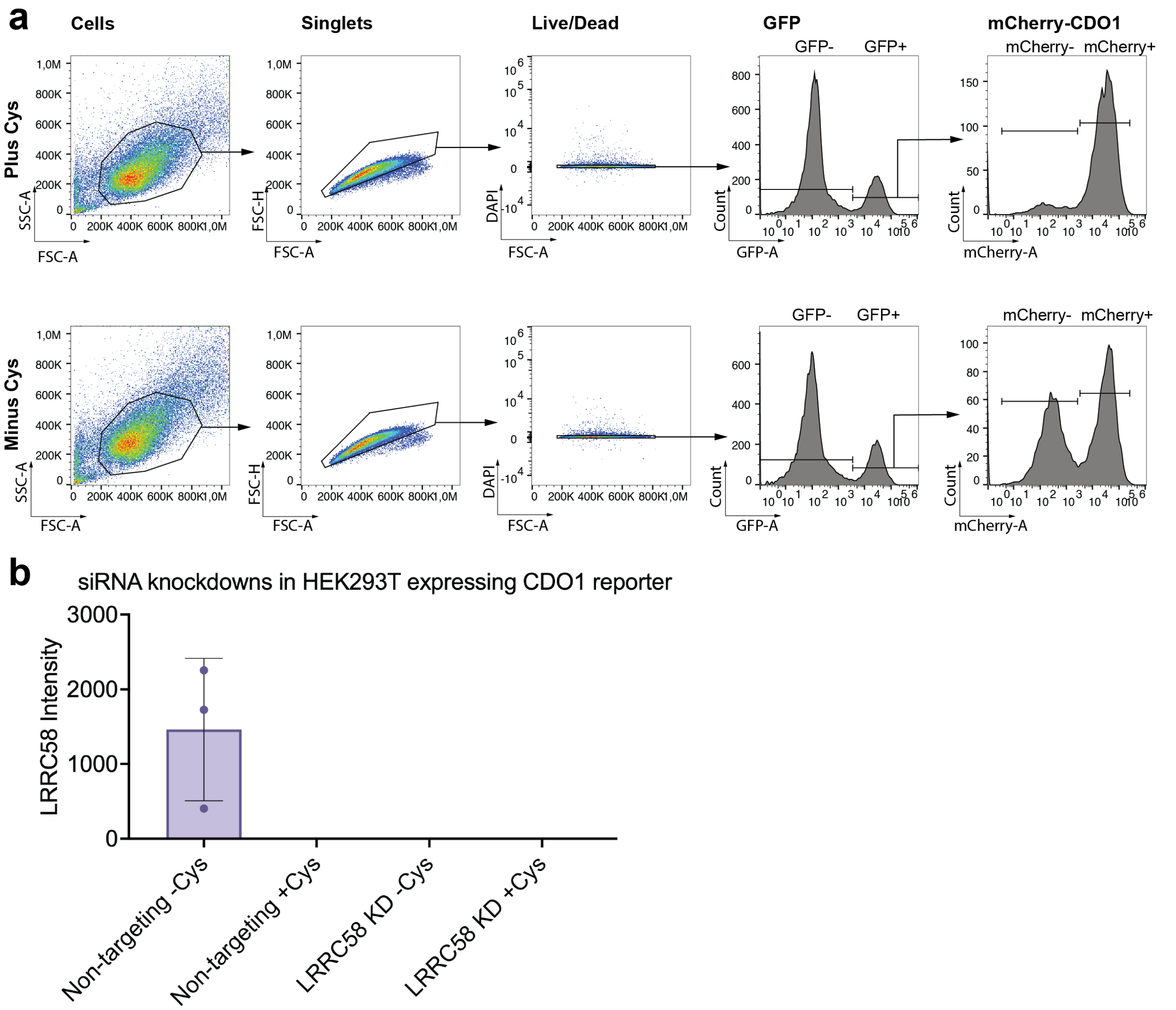

Supplementary Figure 3. CDO1 stability reporter array. (a) Representative gating strategy for HEK293T CDO1 reporter cells analyzed in FlowJo. Cells were first gated using FSC-A/SSC-A to exclude debris, followed by doublet discrimination using FSC-A/FSC-H. Live cells were identified based on DAPI negativity. A histogram of the live-cell population was then used to gate GFP-positive (GFP^+) reporter cells, within which mCherry-positive ($mCherry^+$) and mCherry-negative ($mCherry^-$) subpopulations were defined. Changes in mCherry-signal were subsequently used to assess CDO1 degradation. (b) LRRC58 knockdown (KD) efficiency was determined through analysis of the total proteome of HEK293T cells expressing CDO1 reporter in the absence and presence of extracellular cysteine (-/+ Cys), as compared to the non-targeting control. Source data provided as Source Data file.

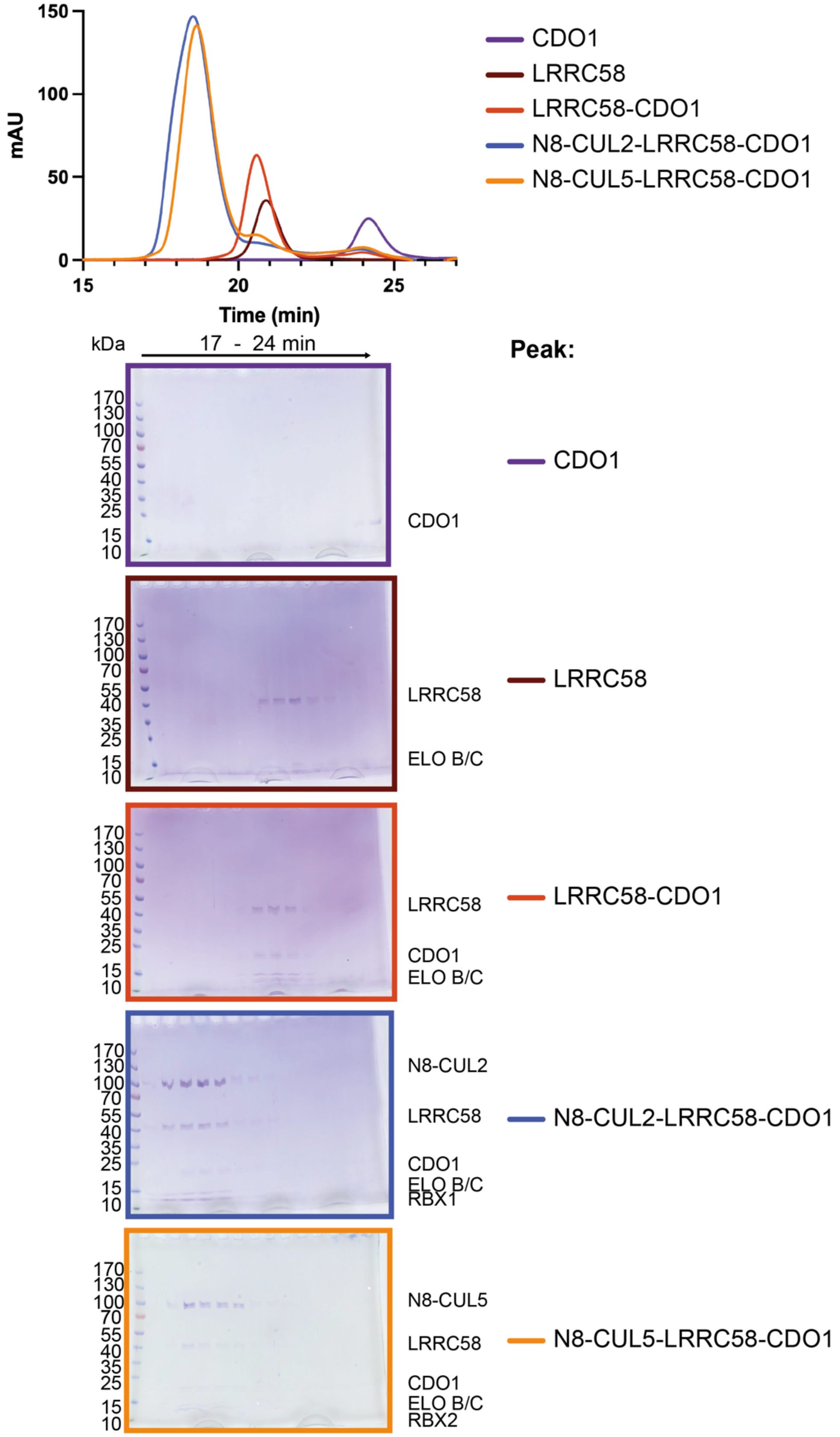

Supplementary Figure 4. LRRC58-EloB/C-CDO1 complex stably interacts with neddylated CUL2-RBX1 and neddylated CUL5-RBX2. Graph showing UV absorbance versus Superdex 200 Increase 3.2/300 gel filtration column retention time for the indicated protein samples that had been mixed stoichiometrically (5 µM) prior to injection. Shifts in

the peak retention time for substrate receptor-containing CRL2 and CRL5 complexes compared to traces for individual sub-components, indicating complex formation. Coomassie stained SDS-PAGE gels (bordered in colors corresponding to the chromatograms shown above) show the protein components from the indicated gel filtration fractions.

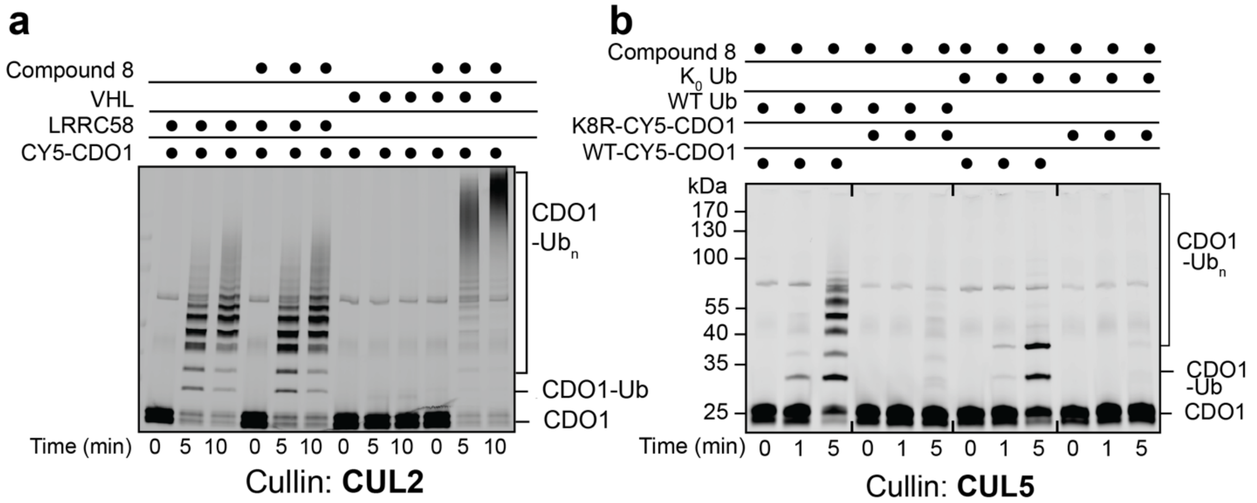

Supplementary Figure 5. The molecular glue degrader, Compound 8, promotes CDO1 ubiquitylation to a greater extent than the native pathway and selective CDO1 Lys targeting is also observed with LRRC58-CUL5. (a) In vitro reconstituted ubiquitylation reactions containing Cy5-labeled CDO1 with neddylated CUL2-RBX1 and in the absence or presence of LRRC58-EloB/C (native pathway) or VHL-EloB/C (degrader pathway) and compound 8 (Cmpd8) or DMSO control. Assay is performed with wild-type ubiquitin. Cmpd8 does not increase CDO1 ubiquitylation with LRRC58-EloB/C as substrate receptor but massively increases CDO1 ubiquitylation with VHL-EloB/C. Fluorescence scan is representative of n=3 technical replicates. (b) In vitro reconstituted assays comparing Cy5-labeled WT and K8R CDO1 ubiquitylation in the presence of neddylated LRRC58-CUL5 and Cmpd8. Assay performed with both wild-type ubiquitin (WT Ub) and a lysine-less ubiquitin (K_0-Ub) that cannot form chains. The efficiency of K8R CDO1 ubiquitylation is significantly lower than WT CDO1. Source data provided as Source Data file.

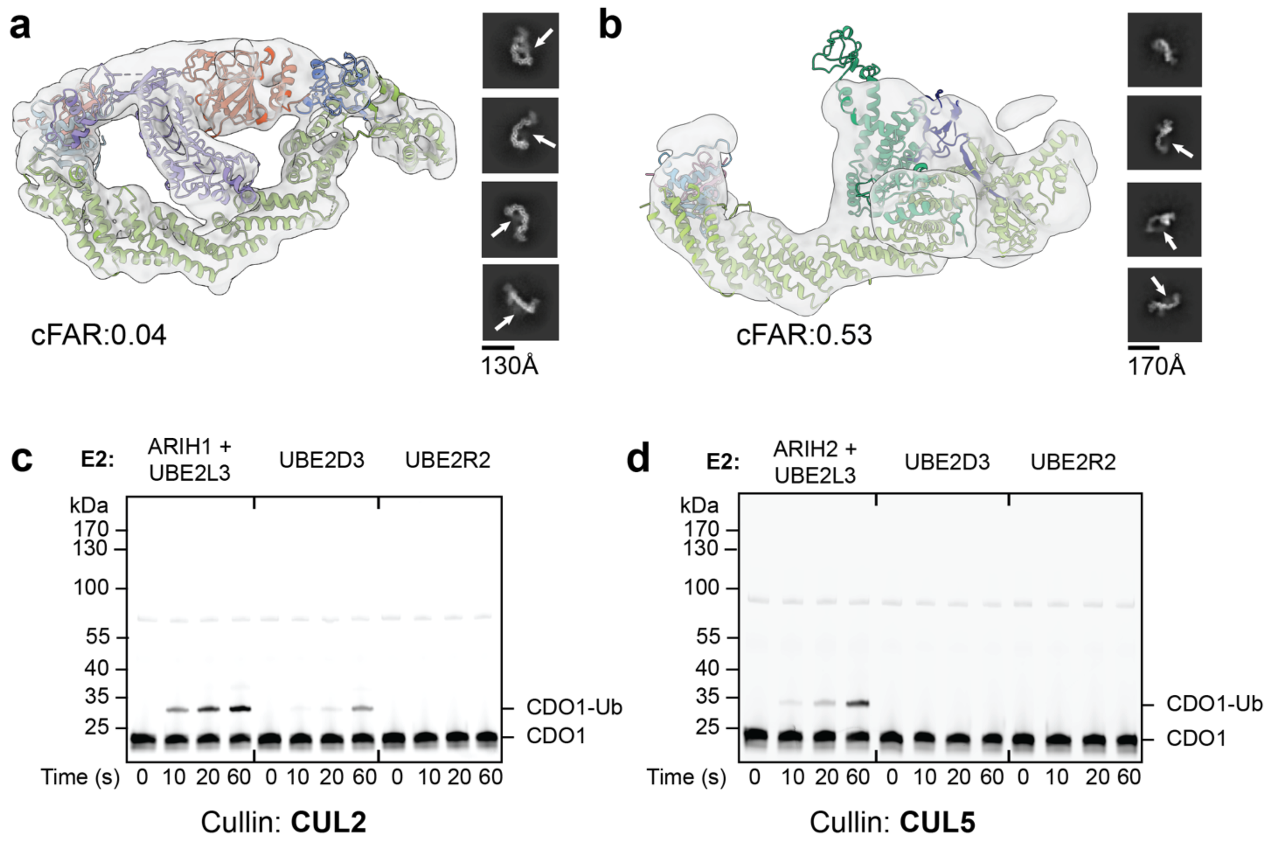

Supplementary Figure 6. Low resolution cryo-EM reconstructions of CRL-LRRC58-CDO1 complexes and comparison of CDO1 ubiquitylation by CRL partner ubiquitylation enzymes. (a) Cryo-EM density (transparent grey) of the complex of LRRC58-CDO1 with CUL2-RBX1 with CUL2 (light green), EloB (pink), EloC (light blue), LRRC58 (purple), CDO1 (orange), and RBX1 (navy) rigid-body fit into the density. cFAR score quantifies the level of preferred orientation which contributed to the low resolution. Representative 2D classes also highlight the overrepresentation of cullin scaffold while simultaneous lack of LRRC58-CDO1 density (white arrows). (b) Cryo-EM density (transparent grey) of the complex of LRRC58-CDO1 with CUL5-RBX2 with CUL5 (light green), EloB (pink), EloC (light blue), ARIH2 (green), and RBX2 (navy) rigid-body fit into the density. LRRC58-CDO1 could not be resolved. Representative 2D classes also highlight the of LRRC58-CDO1 density (white arrows) and cFAR score quantifies the level of preferred orientation. (c) In vitro reconstitution of CDO1 ubiquitylation by neddylated LRRC58-CUL2 with the use of either ARIH1 and UBE2L3, UBE2D3 alone, or UBE2R2 alone as the partner E2 ubiquitylation enzymes. Assays were performed with lysine-less ubiquitin (K_0-Ub) that cannot form chains. (d) Same as in (c) except with the use of neddylated LRRC58-CUL5. The use of ARIH-family enzymes result in the most efficient CDO1 ubiquitylation. Source data provided as Source Data file.

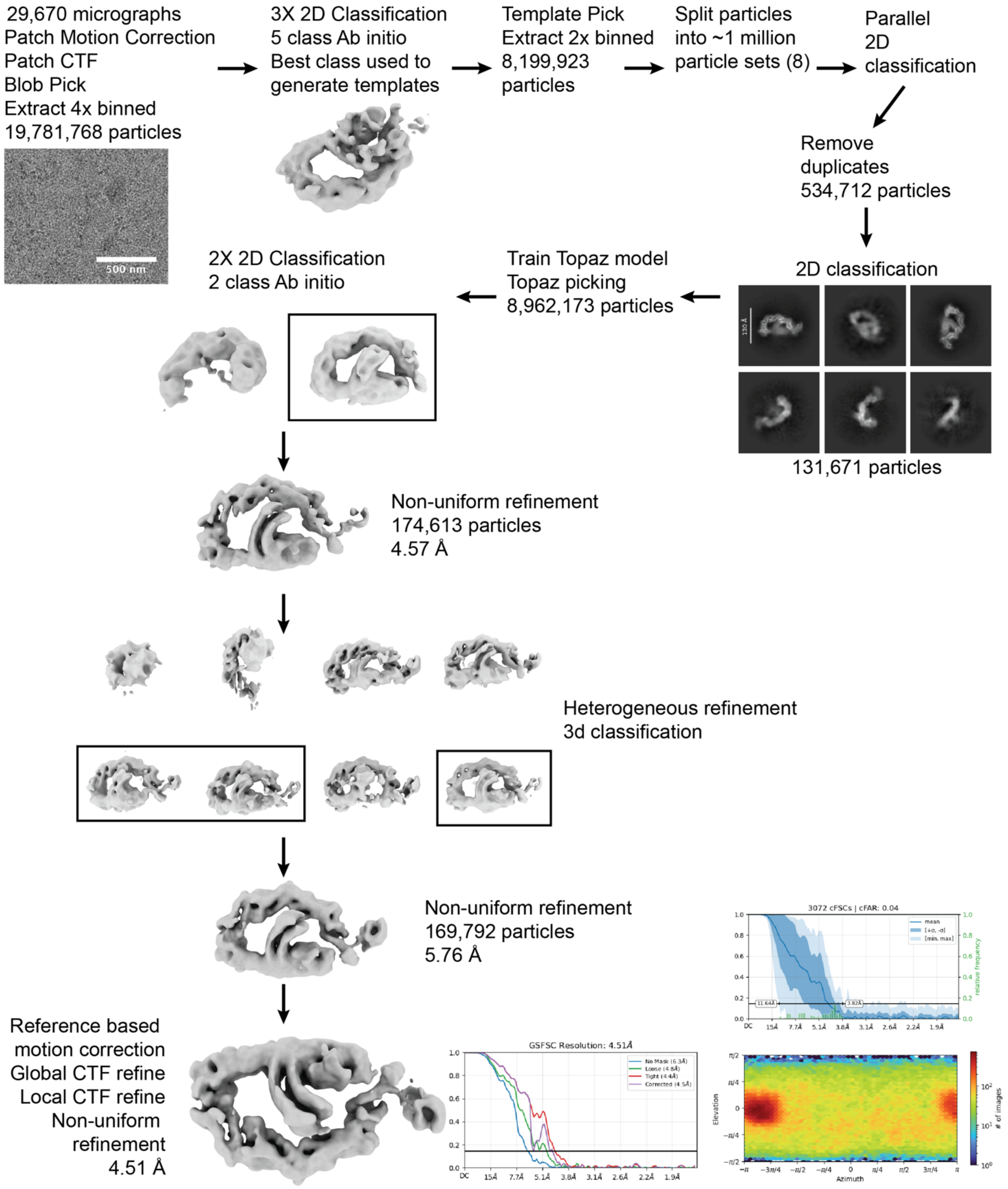

Supplementary Figure 7. Cryo-EM processing scheme for low resolution CDO1-LRRC58 CRL2 complex. Data processed in CryoSPARC v4.7.1 yielded a 3D reconstruction with a resolution of 4.51 Å (as determined by the gold-standard Fourier shell correlation of 0.143, shown). Orientation distribution plot of particles from final refinement also shown.

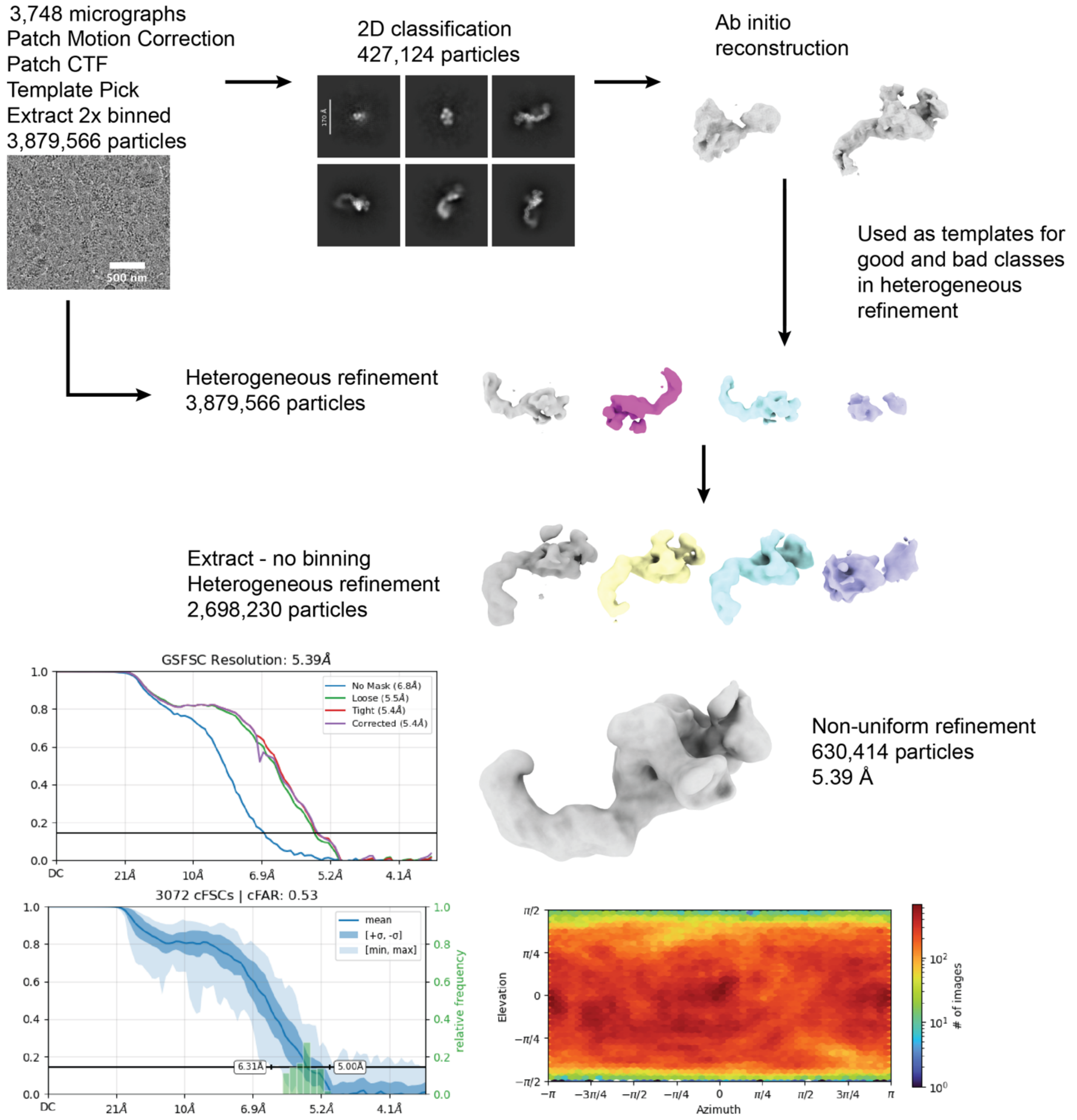

Supplementary Figure 8. Cryo-EM processing scheme for low resolution CDO1-LRRC58 CRL5 complex. Data processed in CryoSPARC v4.7.1 yielded a 3D reconstruction with a resolution of 5.39 Å (as determined by the gold-standard Fourier shell correlation of 0.143, shown). Orientation distribution plot of particles from final refinement also shown.

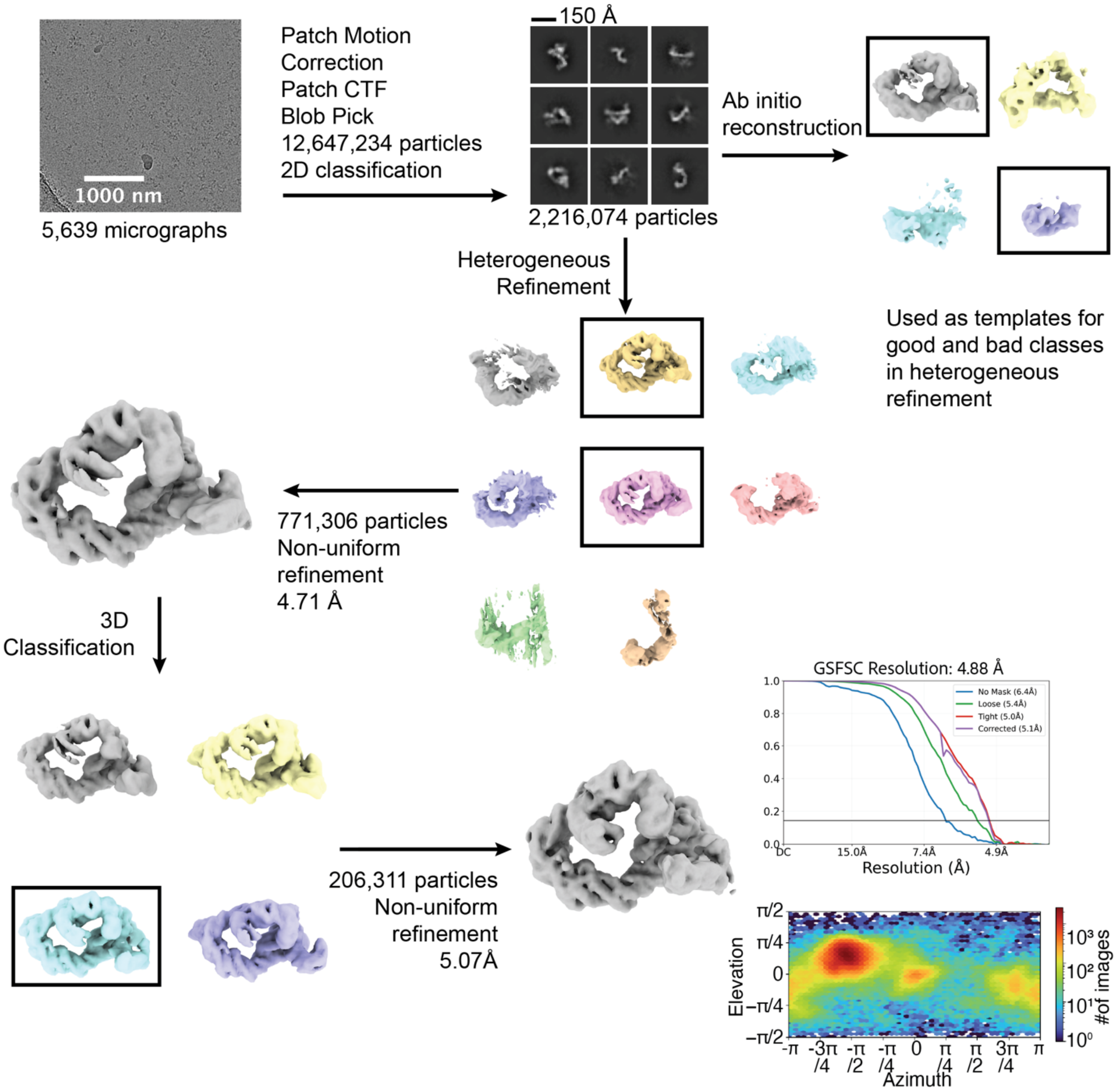

Supplementary Figure 9. Cryo-EM processing scheme for screening dataset for sample representing CDO1 ubiquitylation by LRRC58 with neddylated CUL2-RBX1-ARIH1. Data processed in CryoSPARC v4.7.1 yielded a 3D reconstruction with a resolution of 5.07 Å (as determined by the gold-standard Fourier shell correlation of 0.143, shown). Orientation distribution plot of particles from final refinement also shown. This volume was used as input for Heterogeneous Refinement in Supplementary Fig. 10.

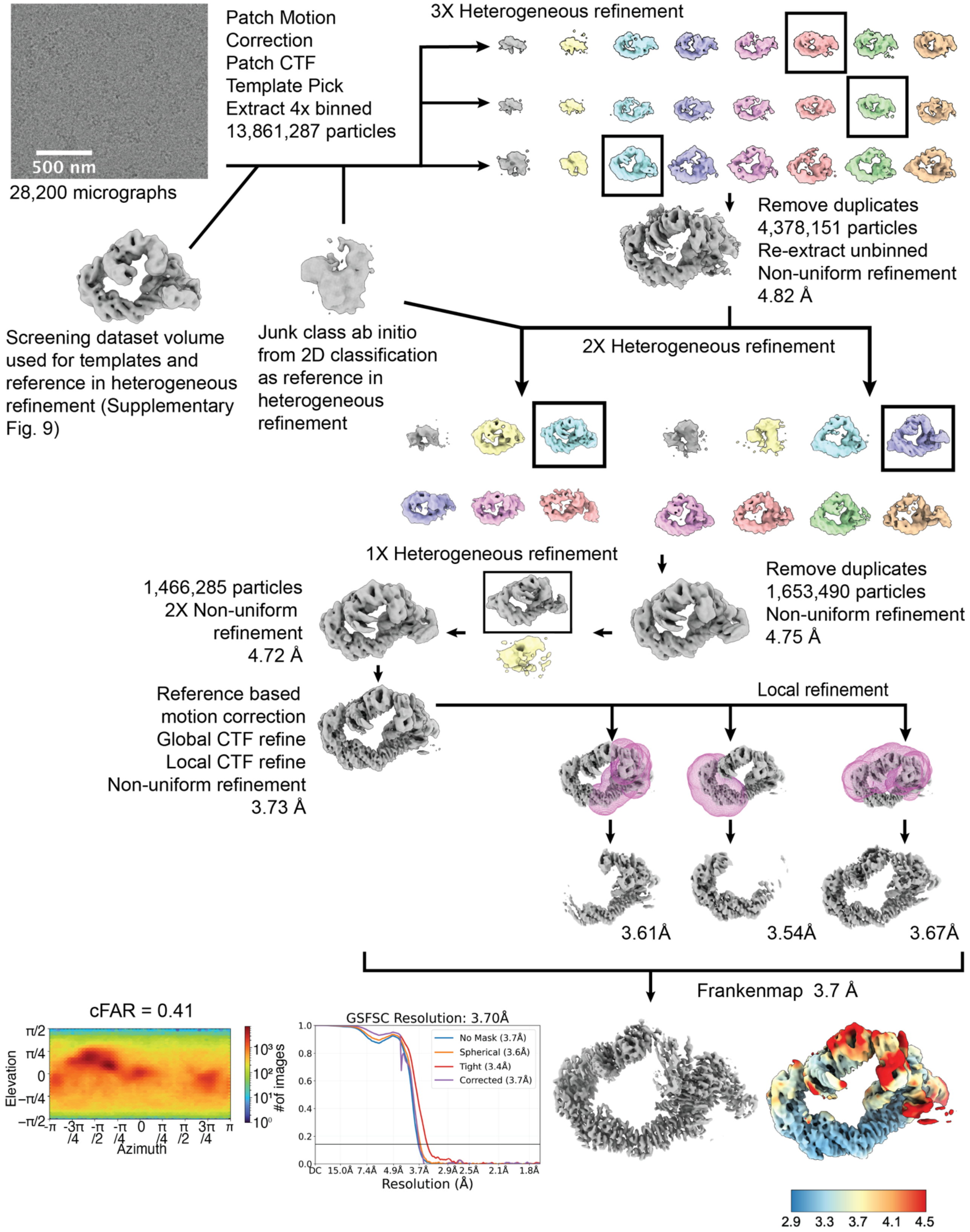

Supplementary Figure 10. Cryo-EM processing scheme for sample representing CDO1 ubiquitylation by LRRC58 with neddylated CUL2-RBX1-ARIH1. Data processed in CryoSPARC v4.7.1 yielded a consensus 3D reconstruction with a resolution of 3.73 Å, and three locally refined maps with resolutions of 3.61 Å, 3.54 Å, and 3.67 Å. Locally refined maps were combined with the consensus map to create a 3.7 Å composite map (as determined by the gold-standard Fourier shell correlation of 0.143, shown) using

Frankenmap (Warp v1.9.0). The orientation distribution plot of particles from consensus refinement is also shown. Local resolution mapped onto the consensus refinement volume is also shown.

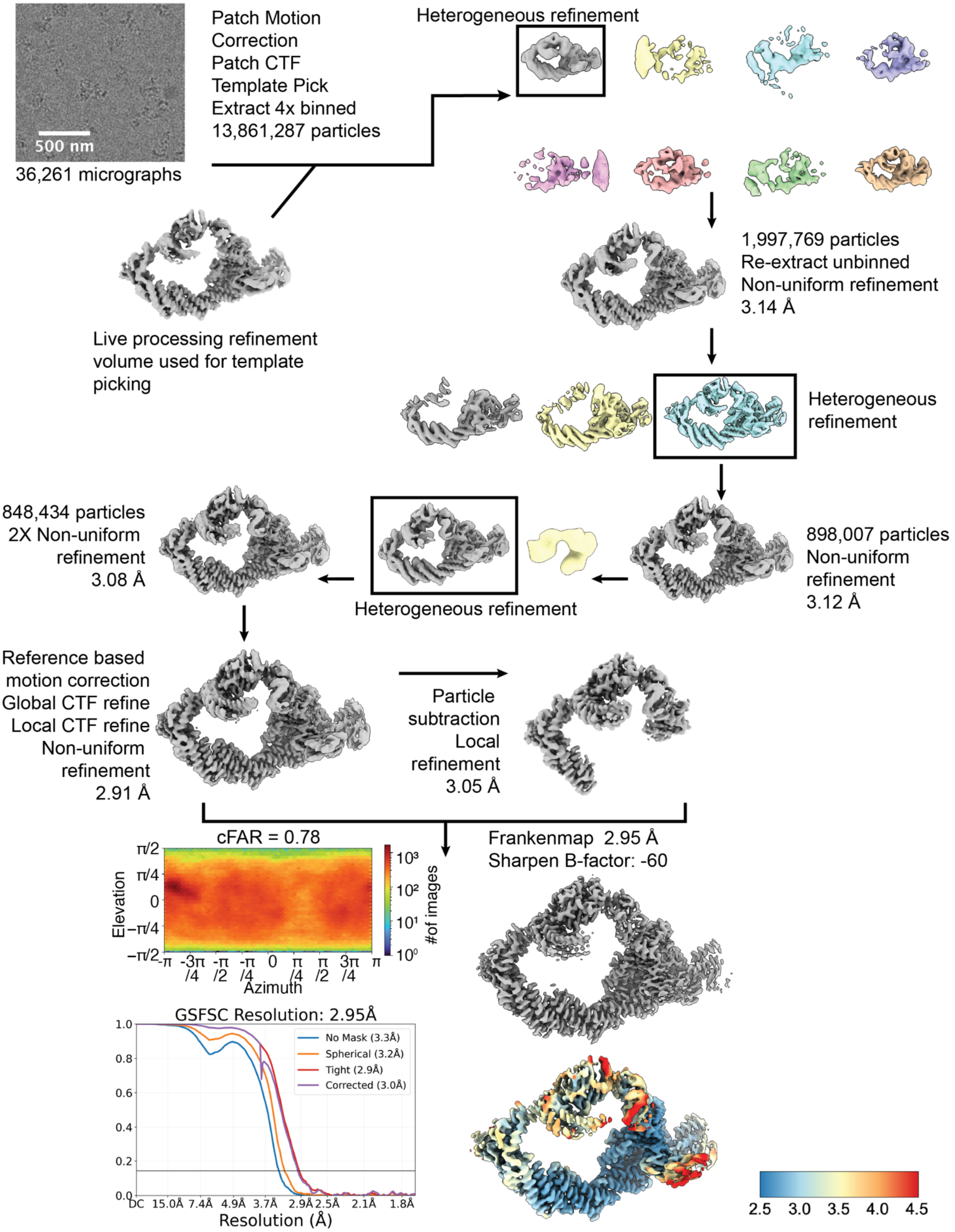

Supplementary Figure 11. Cryo-EM processing scheme for sample representing - CDO1 ubiquitylation by LRRC58 with neddylated CUL5-RBX2-ARIH2. Data processed in CryoSPARC v4.7.1 yielded a consensus 3D reconstruction with a resolution of 2.91 Å, and a particle subtracted and locally refined map with a resolution of 3.05 Å. The particle subtracted map was combined with the consensus map to create a 2.95 Å composite map (as determined by the gold-standard Fourier shell correlation of 0.143, shown) using

Frankenmap (Warp v1.9.0). The orientation distribution plot of particles from consensus refinement is also shown. Local resolution mapped onto the consensus refinement volume is also shown

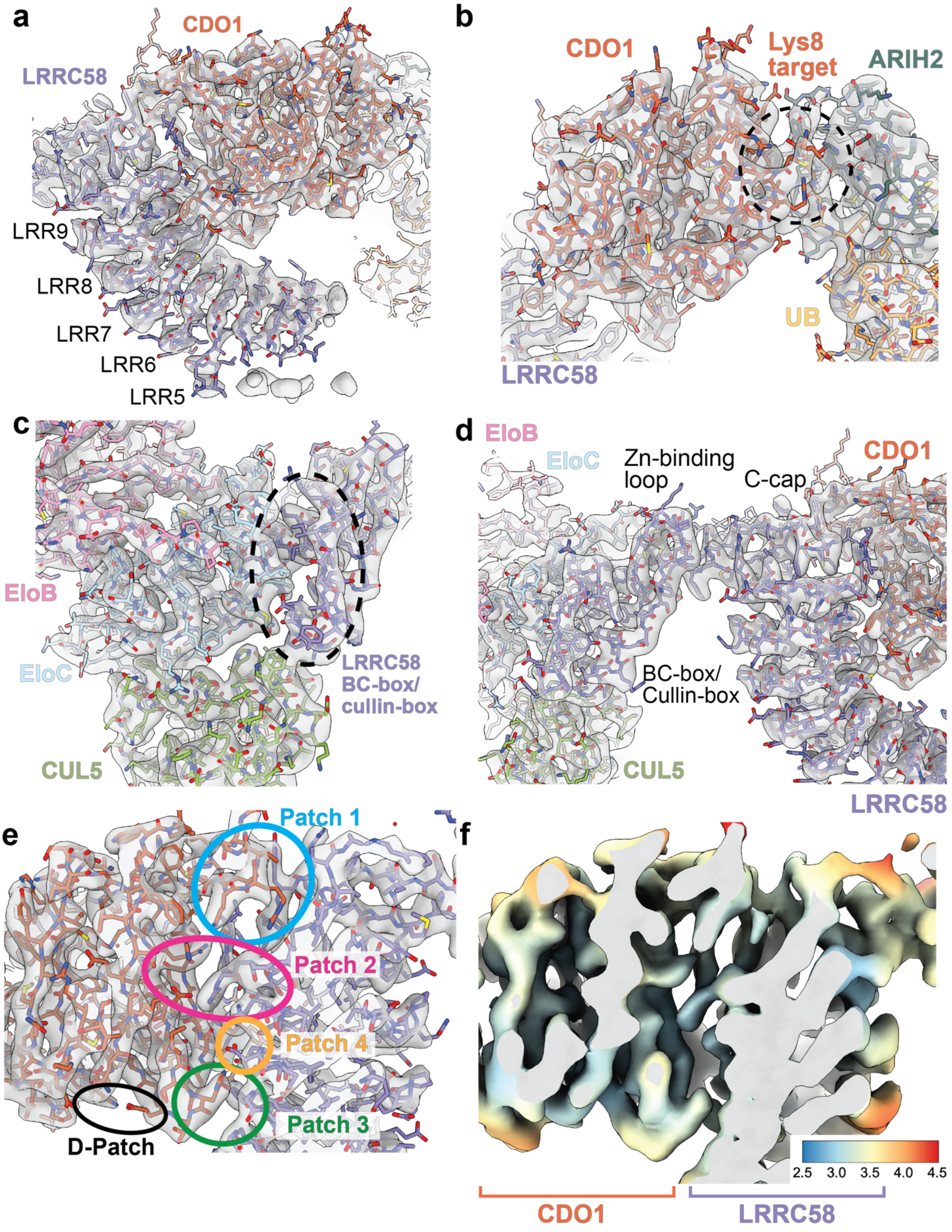

Supplementary Figure 12. Example fits of the structure representing CDO1 ubiquitylation by LRRC58 with neddylated CUL5-RBX2-ARIH2 into the cryo-EM reconstruction. Atomic model representing the ubiquitylation intermediate in the CDO1-LRRC58 complex with CUL5-RBX2-ARIH2 shown in stick representation and fit in the corresponding composite cryo-EM map (transparent grey). (a) Overview of LRRC58 (purple) interaction with CDO1 (orange), highlighting the positions of the LRR regions that had sufficient electron density to enable their modeling. (b) Zoomed view of CDO1

(orange) highlighting the ARIH2 Rcat domain (LRRC58 in purple, ARIH2 in dark teal, Ub in gold). CDO1 Lys8 target position, here replaced by a Cys-substitution to enable three-way cross-linking between ARIH2, ubiquitin and CDO1 (dashed black circle), mimicking the high energy ubiquitylation transition state intermediate. (c) Molecular details of the interfaces mediated EloC-LRRC58-CUL5 interaction (EloB inpink, EloC in light blue, CUL5 in light green, and LRRC58 in purple). LRRC58's BC- and Cullin-boxes are identified by the dashed circle. (d) Same as (c) but with an expanded view including CDO1 (orange) and LRRC58's C-cap and Zn binding loop regions. (e) Zoomed view highlighting the LRRC58-CDO1 intermolecular interface and various subregions (patch 1-blue, patch 2-pink, patch 3-green, patch 4-yellow, and D-patch). (f) Local resolution estimation mapped onto the density show in (e).

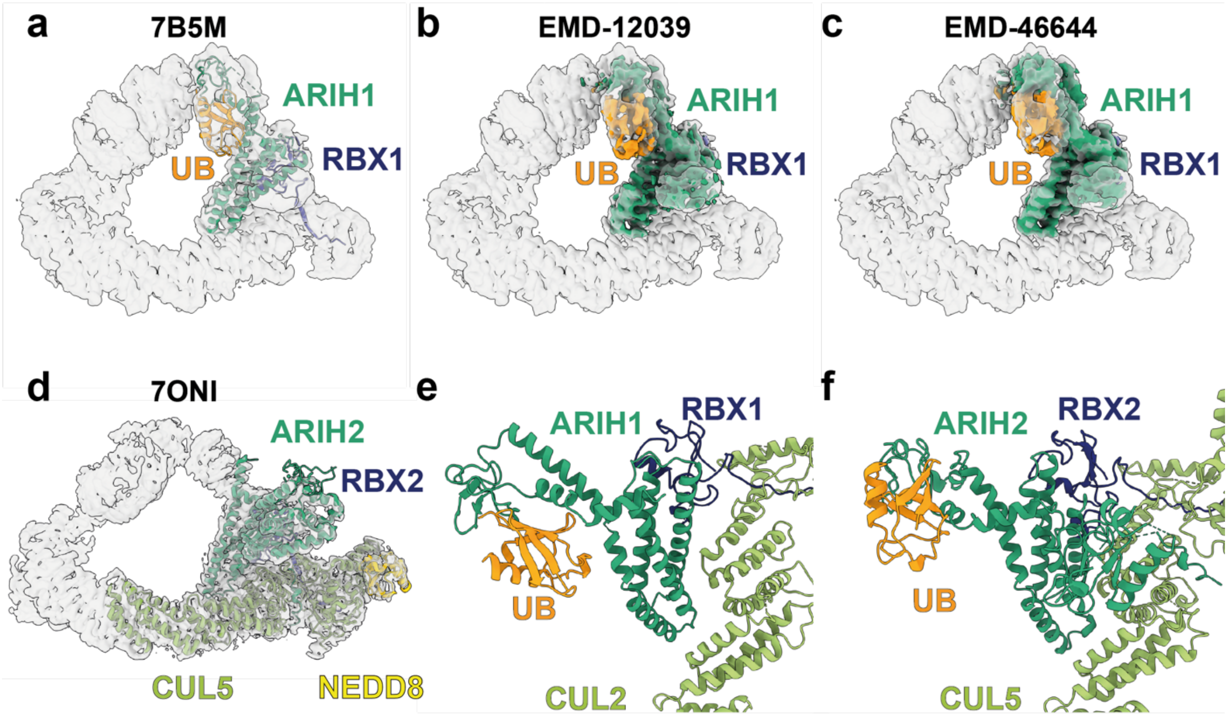

Supplementary Figure 13. RBX1-based CRLs shows distinct catalytic arrangement from RBX2-based CRLs. (a) The RBX1-ARIH1~ubiquitin portion of a CUL1 CRL structure (RBX1 in navy, ubiquitin in gold, and ARIH1 in green, PDB: 7B5M) fit into the composite map representing CDO1 ubiquitylation by LRRC58 with neddylated CUL2-RBX1-ARIH1 (transparent grey). (b) Same as in (a), but the RBX1-ARIH1~ubiquitin region of a CUL1 CRL cryo-EM map (EMD-12039). (c) Same as in (a) and (b), but the RBX1-ARIH1~ubiquitin region of a CUL2 CRL cryo-EM map (EMD-46644). (d) The CUL5 C-terminal domain (light green), ARIH2 (green), and NEDD8 (yellow) which did not have ubiquitin at the ARIH2 active site (PDB: 7ONI) fit into the composite map representing CDO1 ubiquitylation by LRRC58 with neddylated CUL5-RBX2-ARIH2 (transparent grey). (e) The RBX1-ARIH1~ubiquitin portion of the model representing CDO1 ubiquitylation by LRRC58 with neddylated CUL2-RBX1-ARIH1, aligned on the ARIH2 domain of the RBX2-ARIH2~ubiquitin portion in the structure representing CDO1 ubiquitylation by LRRC58 with neddylated CUL5-RBX2-ARIH2 in panel (f). The catalytic region is oriented differently between the two CRL complexes.

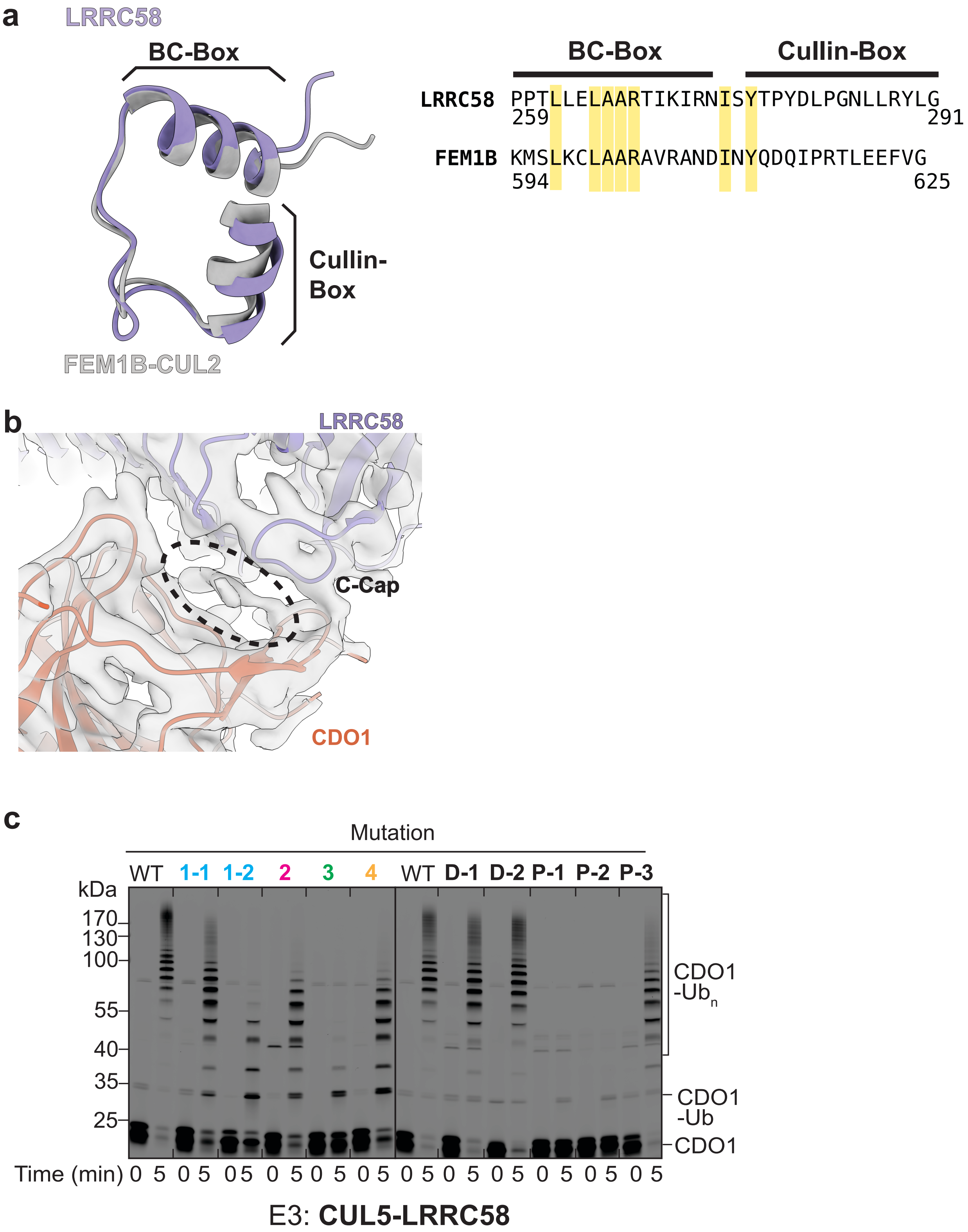

Supplementary Figure 14. CUL2/5-dependent substrate receptors share conserved BC-box structures. (a) Ribbon diagrams showing structural alignment of the BC- and Cullin-Boxes from the LRRC58 with CUL2-dependent substrate receptor FEM1B's BC- and Cullin-Boxes (grey, PDB: 8WQH). Amino acid sequences occupying structural equivalent positions from LRRC58 and FEM1C are aligned (identical residues are highlighted in yellow). (b) The LRRC58 (purple)-CDO1 (orange) interface from the structure representing CDO1 ubiquitylation by LRRC58 with neddylated CUL5-RBX2-

ARIH2. Cryo-EM density is shown in transparent grey. Ambiguous density (highlighted by the black dotted oval) is observed between CDO1 and the C-cap of LRRC58. (c) In vitro reconstituted assays comparing WT and mutant Cy5-CDO1 ubiquitylation by neddylated LRRC58-CUL5. Mutations only in patch interfaces, including patient variants, reduced CDO1 ubiquitylation. Fluorescence scans are representative of n=2 technical replicates. Source data provided as Source Data file.

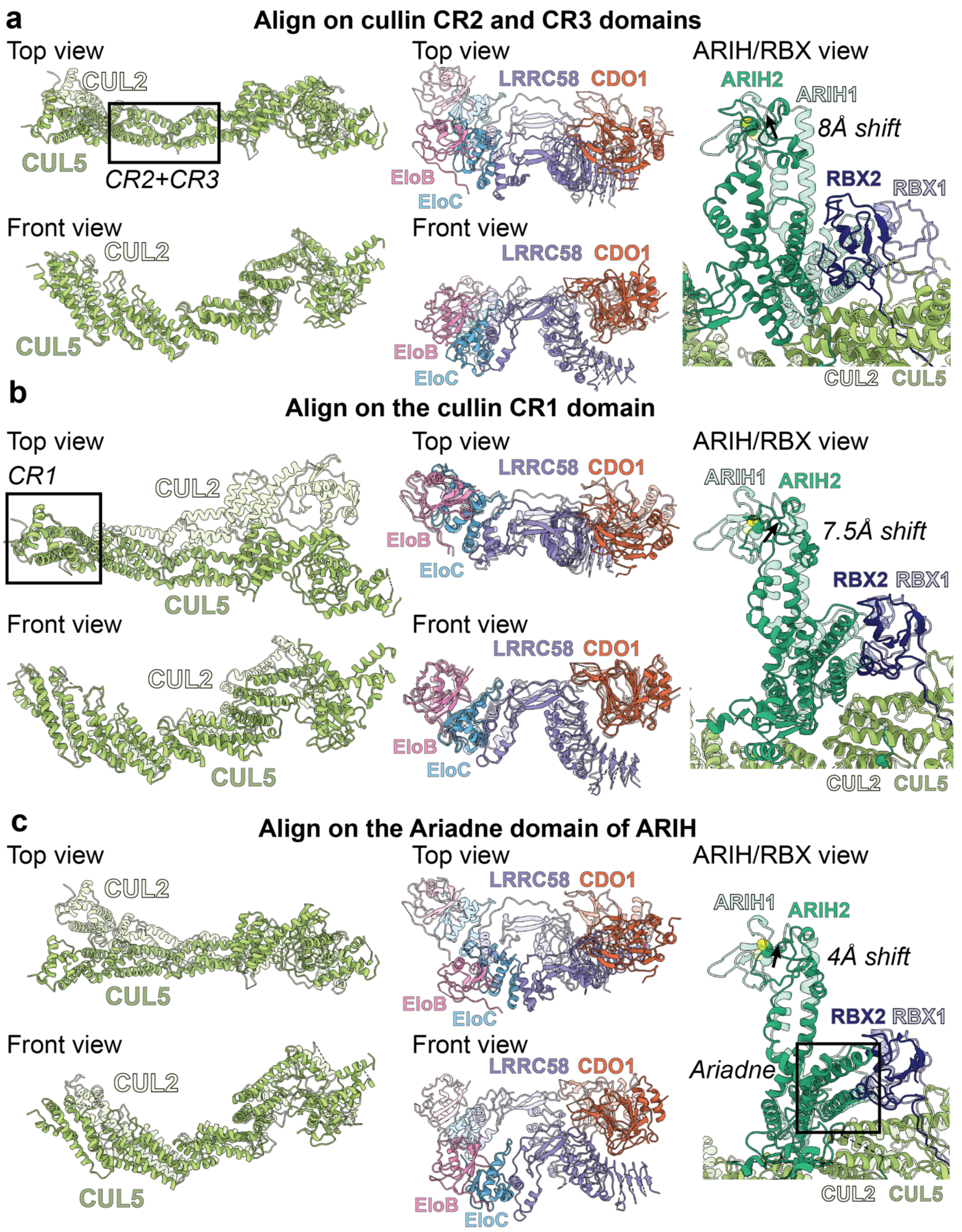

Supplementary Figure 15. Conformational flexibility of LRRC58-CDO1 in complex with either neddylated CUL2 or CUL5 and their respective ubiquitin-carrying enzymes. (a) The complexes of LRRC58-CDO1 with either neddylated CUL2-RBX1-ARIH1 or neddylated CUL5-RBX2-ARIH2 were aligned upon the cullin repeat (CR) domains 2 and 3 (highlighted with a black square). In each panel, different subunits of the complex are shown alone, for clarity. On the right, a top and front view of the the CUL2 (transparent green) and CUL5 (light green) scaffold are displayed and show similar

conformations. In the middle, only EloB/C-LRRC58-CDO1 (EloB in pink, EloC in light blue, LRRC58 in purple, CDO1 in orange, all colors in the CUL2 complex are transparent), are shown in both a top and front view. A specific ARIH/RBX view is shown at left (ARIH2 in green, ARIH1 in transparent green, RBX2 in navy, RBX1 in transparent navy, CUL5 in green, CUL2 in transparent green) with the catalytic residue (C310 in ARIH2, C357 in ARIH1) indicated in spheres. An ~8 Å shift in this residue is seen. (b) Same as in (a), except aligning on the CR1 domain. On the right, a C-terminal shift of the cullins is evident. This equates to a small shift in CDO1 placement, as seen in the middle panels. The ARIH active site residues have ~7.5 Å shift in this alignment. (c) Same as in (a) and (b), except aligning on the Ariadne domain of ARIH1 and 2. On the left an N-terminal shift of the cullin scaffold can be seen in the top view. In the middle, this shift is also evident by the shift in EloB/C locations. Only a ~4 Å shift of ARIH catalytic residues is seen in the leftmost view.

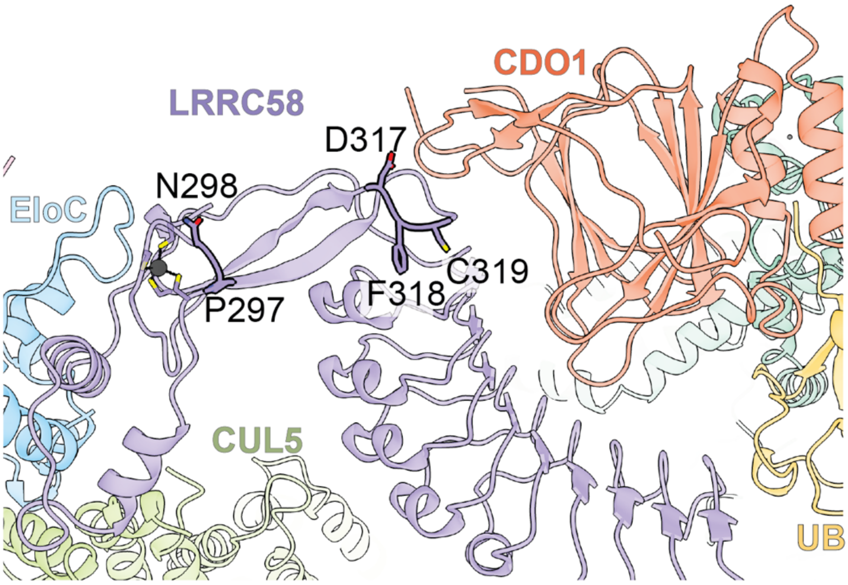

Supplementary Figure 16. Potential LRRC58 cysteine sensing residues are located close to the Zn-binding loop or in the C-cap of LRRC58. Mapping of LRRC58 residues (in opaque sticks, outlined in black), identified by saturation mutagenesis by Ramage et al., 2025, that may function as cysteine sensors. Residues are shown on the structure representing CDO1 ubiquitylation by LRRC58 with neddylated CUL5-RBX2-ARIH2 (in transparent ribbons; LRRC58 in purple, EloC in light blue, CDO1 in orange, CUL5 in light green, Ub in gold, ARIH2 in green). The "cysteine sensor" residues are either in the C-cap region, or are located close to the LRRC58 Zn-binding loop. Residues L330, M365, and G371 were identified but are not visualized in our structure.
