# Supplementary material for: Cysteine availability tunes ubiquitin signaling via inverse stability of LRRC58 E3 ligase and its substrate CDO1
Source: Nat Commun. 2026 May 7;17:4196. doi: 10.1038/s41467-026-72524-3 (PMC13156300; doi:10.1038/s41467-026-72524-3)
Supplement: Supplementary file 2 — Description of Additional Supplementary Files [file 41467_2026_72524_MOESM2_ESM.pdf]

Description of Additional Supplementary Files

File name: Supplementary Data 1

Description: Mass spectrometry data tables for all datasets in this study. Included are DIA-NN protein group reports, directLFQ normalized intensities, and list of CRL substrate binding modules and the cullin they are assumed to primarily associate with.

File name: Supplementary Data 2

Description: Sequences (FASTA format) of proteins used as input in HT-Colabfold with LRRC58, EloB, and EloC sequences (not provided) used as bait.
