# Supplementary material for: Cysteine availability tunes ubiquitin signaling via inverse stability of LRRC58 E3 ligase and its substrate CDO1
Source: Nat Commun. 2026 May 7;17:4196. doi: 10.1038/s41467-026-72524-3 (PMC13156300; doi:10.1038/s41467-026-72524-3)
Supplement: Supplementary file 5 — Reporting Summary [file 41467_2026_72524_MOESM5_ESM.pdf]

nature portfolio

Corresponding author(s): Brenda A. Schulman

Last updated by author(s): Mar 18, 2026

Reporting Summary

Nature Portfolio wishes to improve the reproducibility of the work that we publish. This form provides structure for consistency and transparency in reporting. For further information on Nature Portfolio policies, see our Editorial Policies and the Editorial Policy Checklist.

Statistics

For all statistical analyses, confirm that the following items are present in the figure legend, table legend, main text, or Methods section.

n/a	Confirmed	
☐	☒	The exact sample size (n) for each experimental group/condition, given as a discrete number and unit of measurement
☐	☒	A statement on whether measurements were taken from distinct samples or whether the same sample was measured repeatedly
☐	☒	The statistical test(s) used AND whether they are one- or two-sided<br>*Only common tests should be described solely by name; describe more complex techniques in the Methods section.*
☒	☐	A description of all covariates tested
☒	☐	A description of any assumptions or corrections, such as tests of normality and adjustment for multiple comparisons
☐	☒	A full description of the statistical parameters including central tendency (e.g. means) or other basic estimates (e.g. regression coefficient) AND variation (e.g. standard deviation) or associated estimates of uncertainty (e.g. confidence intervals)
☐	☒	For null hypothesis testing, the test statistic (e.g. F, t, r) with confidence intervals, effect sizes, degrees of freedom and P value noted<br>*Give P values as exact values whenever suitable.*
☒	☐	For Bayesian analysis, information on the choice of priors and Markov chain Monte Carlo settings
☒	☐	For hierarchical and complex designs, identification of the appropriate level for tests and full reporting of outcomes
☒	☐	Estimates of effect sizes (e.g. Cohen's d, Pearson's r), indicating how they were calculated

Our web collection on statistics for biologists contains articles on many of the points above.

Software and code

Policy information about availability of computer code

Data collection	Cryo-EM data acquisition: SerialEM v4.2.1; Gel & blot imaging: Amersham ImageQuant 800; Fluorescent gel scanning: Amersham Typhoon; Mass Spectrometry data acquisition: Bruker Compass HyStar v6.2
Data analysis	Cryo-EM data processing: CryoSparc v4.7.1 and v4.7.1+250814 (Patch), Warp 1.9.0<br>Structure Analysis and Visualization: ChimeraX v1.9, DeepEMhancer version 2020.09.07 (https://github.com/rsanchezgarc/deepEMhancer);<br>Model Building & Refinement: COOT v0.9.8.96, Phenix.refine v1.21.1, AlphaFold3;<br>Biochemical Data Analysis: ImageJ 1.54, GraphPad Prism 10.6.1; Figure Generation: Adobe Illustrator 2026<br>Mass spectrometry data analysis: DIA-NN v1.9.2, Perseus v.2.0.9.0, DirectLFQ v0.3.2<br>Other Analysis: HT-Colabfold (https://gitlab.com/BrenneckeLab/ht-colabfold)<br>Flow Cytometry: FlowJo 10.8.2<br>RNA Seq: FastQC v.0.11.7, STAR aligner v. 2.7.10b, featureCounts tool v. 2.0.4, DESeq2 package in the R statistical environment (v. 4.3.2), bases2fastq software (Element)<br>All software used was either available commercially (CryoSPARC, GraphPad Prism, Adobe Illustrator) or as open source (others).

For manuscripts utilizing custom algorithms or software that are central to the research but not yet described in published literature, software must be made available to editors and reviewers. We strongly encourage code deposition in a community repository (e.g. GitHub). See the Nature Portfolio guidelines for submitting code & software for further information.

Data

Policy information about availability of data

All manuscripts must include a data availability statement. This statement should provide the following information, where applicable:

- Accession codes, unique identifiers, or web links for publicly available datasets
- A description of any restrictions on data availability
- For clinical datasets or third party data, please ensure that the statement adheres to our policy

The structure data for LRRC58-EloB/C-CDO1 in complex with NEDD8-CUL5-Ub-RBX2-ARIH2 are available from the RCSB-PDB and EMDB with the identifiers; 9T7V [http://doi.org/10.2210/pdb9T7V/pdb], EMD-55658 [https://www.ebi.ac.uk/pdbe/entry/emdb/EMD-55658], EMD-55659 [https://www.ebi.ac.uk/pdbe/entry/emdb/EMD-55659], EMD-55660 [https://www.ebi.ac.uk/pdbe/entry/emdb/EMD-55660]. The cryo-EM volumes for LRRC58-EloB/C-CDO1 in complex with NEDD8-CUL2-Ub-RBX1-ARIH1 are available from the EMDB with the identifiers; EMD-55652 [https://www.ebi.ac.uk/pdbe/entry/emdb/EMD-55652], EMD-55653 [https://www.ebi.ac.uk/pdbe/entry/emdb/EMD-55653], EMD-55654 [https://www.ebi.ac.uk/pdbe/entry/emdb/EMD-55654], EMD-55655 [https://www.ebi.ac.uk/pdbe/entry/emdb/EMD-55655], EMD-55656 [https://www.ebi.ac.uk/pdbe/entry/emdb/EMD-55656]. The mass spectrometry proteomics data have been deposited to the ProteomeXchange Consortium via the PRIDE partner repository with the identifier; PXD071631 [http://proteomecentral.proteomexchange.org/cgi/GetDataset?ID=PXD071631]. The RNA-Seq data have been deposited in NCBI's Gene Expression Omnibus and are accessible through GEO Series accession number GSE309930 [https://www.ncbi.nlm.nih.gov/geo/query/acc.cgi?acc=GSE309930].

Research involving human participants, their data, or biological material

Policy information about studies with human participants or human data. See also policy information about sex, gender (identity/presentation), and sexual orientation and race, ethnicity and racism.

Reporting on sex and gender	No research involving human participants has been performed.
Reporting on race, ethnicity, or other socially relevant groupings	No research involving human participants has been performed.
Population characteristics	No research involving human participants has been performed.
Recruitment	No research involving human participants has been performed.
Ethics oversight	No research involving human participants has been performed.

Note that full information on the approval of the study protocol must also be provided in the manuscript.

Field-specific reporting

Please select the one below that is the best fit for your research. If you are not sure, read the appropriate sections before making your selection.

☒ Life sciences ☐ Behavioural & social sciences ☐ Ecological, evolutionary & environmental sciences

For a reference copy of the document with all sections, see nature.com/documents/nr-reporting-summary-flat.pdf

Life sciences study design

All studies must disclose on these points even when the disclosure is negative.

Sample size	Sample size calculations were not performed. Generally, we carried out at least three biological replicates for experiments involving cultured cells to ascertain result reproducibility.
Data exclusions	No data were excluded.
Replication	All biochemical experiments were performed (at least) in technical duplicates along with appropriate controls, with successful replication of results in all cases.
Randomization	No grouped samples.
Blinding	Blinding is not relevant to this study.

Reporting for specific materials, systems and methods

We require information from authors about some types of materials, experimental systems and methods used in many studies. Here, indicate whether each material, system or method listed is relevant to your study. If you are not sure if a list item applies to your research, read the appropriate section before selecting a response.

Materials & experimental systems

n/a	Involved in the study	
☐	☒	Antibodies
☐	☒	Eukaryotic cell lines
☒	☐	Palaeontology and archaeology
☒	☐	Animals and other organisms
☒	☐	Clinical data
☒	☐	Dual use research of concern
☒	☐	Plants

Methods

n/a	Involved in the study	
☒	☐	ChIP-seq
☐	☒	Flow cytometry
☒	☐	MRI-based neuroimaging

Antibodies

Antibodies used

CUL2 (abcam, #ab166917, 1:1000),
CUL5 (abcam, #ab184177, 1:1000),
CDO1 (Proteintech, #12589-1-AP, 1:500),
GAPDH (Cell Signaling Technology, #2118, 1:1000),
Vinculin (Abcam, # ab129002, 1:1000),
anti-rabbit IgG, HRP-linked (Cell Signaling Technology, #7074, 1:2500)

Validation

CUL2 (abcam,# ab166917, 1:1000) - (https://www.abcam.com/products/primary-antibodies/cullin-2cul-2-antibody-epr31042-ab166917.html; reacts with mouse, rat, human protein)
CUL5 (abcam, #ab184177, 1:1000) - (https://www.abcam.com/products/primary-antibodies/cullin-5cul-5-antibody-eprl4725-ab184177.html; binds human, mouse, rat protein),
CDO1 (Proteintech, #12589-1-AP, 1:500) - (https://www.ptglab.com/products/CDO1-Antibody-12589-1-AP.htm?srsltid=AfmBOorxvz1ABf5DYX-skO4LLx4JrsMCHkPtzkn6qTI67AVwUgFiaAO0; 12589-1-AP targets CDO1 in WB, IHC, IF/ICC, IP, ELISA applications and shows reactivity with human, mouse, rat, pig samples)
GAPDH (Cell Signaling Technology, #2118, 1:1000) - (https://www.cellsignal.com/products/primary-antibodies/gapdh-14c10- rabbit-mab/21l8, GAPDH (14(10) Rabbit mAb detects endogenous levels of total GAPDH protein of human, mouse, rat, monkey, bovine, and pig origin)
Vinculin (Abcam, # ab129002, 1:1000) - (https://www.abcam.com/en-us/products/primary-antibodies/vinculin-antibody-epr8185-loading-control-ab129002; Anti-Vinculin antibody [EPR8185] (ab129002) is a rabbit monoclonal antibody detecting Vinculin in Western Blot, Flow Cytometry (Intra), Flow Cytometry, IP, ICC/IF. Suitable for Human, Mouse, Rat)
anti-rabbit IgG, HRP-linked (Cell Signaling Technology, #7074, 1:2500) - (https://www.cellsignal.com/products/secondary-antibodies/anti-rabbit-igg-hrp-linked-antibody/7074; reacts with rabbit protein)

Eukaryotic cell lines

Policy information about cell lines and Sex and Gender in Research

Cell line source(s)

HEK293T (ACC 635) and HeLa (ACC 57) were obtained from Deutsche Sammlung von Mikroorganismen und Zellkulturen (DSMZ). Jurkat (TIB-152), HepG2 (HB-8065) and SKBR3 were purchased from American Type Culture Collection (ATCC).

Authentication

Cell lines were not authenticated.

Mycoplasma contamination

Cell lines were periodically tested for mycoplasm contamination using a PCR mycoplasma detection kit (Applied Biological Materials) with no contamination detected.

Commonly misidentified lines
(See ICLAC register)

No commonly misidentified cell lines were used in this study.

Plants

Seed stocks

Report on the source of all seed stocks or other plant material used. If applicable, state the seed stock centre and catalogue number. If plant specimens were collected from the field, describe the collection location, date and sampling procedures.

Novel plant genotypes

Describe the methods by which all novel plant genotypes were produced. This includes those generated by transgenic approaches, gene editing, chemical/radiation-based mutagenesis and hybridization. For transgenic lines, describe the transformation method, the number of independent lines analyzed and the generation upon which experiments were performed. For gene-edited lines, describe the editor used, the endogenous sequence targeted for editing, the targeting guide RNA sequence (if applicable) and how the editor was applied.

Authentication

Describe any authentication procedures for each seed stock used or novel genotype generated. Describe any experiments used to assess the effect of a mutation and, where applicable, how potential secondary effects (e.g. second site T-DNA insertions, mosiacism, off-target gene editing) were examined.

Flow Cytometry

Plots

Confirm that:

☒ The axis labels state the marker and fluorochrome used (e.g. CD4-FITC).

☒ The axis scales are clearly visible. Include numbers along axes only for bottom left plot of group (a 'group' is an analysis of identical markers).

☒ All plots are contour plots with outliers or pseudocolor plots.

☒ A numerical value for number of cells or percentage (with statistics) is provided.

Methodology

Sample preparation	Cells after treatments were washed with DPBS and trypsinized with TrypLE for single-cell dissociation before resuspension in DPBS-EDTA (containing 2 mM EDTA). Samples were measured on an Attune NxT (ThermoFisher Scientific) flow cytometer with autosampler
Instrument	Attune NxT (ThermoFisher Scientific)
Software	FlowJo 10.8.2
Cell population abundance	The population of single cells based on FSC/SSC was ~50%
Gating strategy	Single cells were identified via FSC-A/SSC-A gating followed by doublet exclusion using FSC-A/FSC-H. Live cells were subsequently gated based on DAPI negativity. GFP-positive cells were then analyzed for mCherry signal. A 488 nm laser with a 530/30 gate was used to measure GFP. A 405 nm laser with a 440/50 gate was used to measure DAPI. A 531 nm laser with a 620/15 gate was used to measure mCherry.

☒ Tick this box to confirm that a figure exemplifying the gating strategy is provided in the Supplementary Information.
