# Supplementary material for: Cysteine availability tunes ubiquitin signaling via inverse stability of LRRC58 E3 ligase and its substrate CDO1
Source: Nat Commun. 2026 May 7;17:4196. doi: 10.1038/s41467-026-72524-3 (PMC13156300; doi:10.1038/s41467-026-72524-3)

Figure 2f

Marker = Thermo Scientific PageRuler Cat. No. 26617

WESTERN BLOT AS IN FIGURE

RAW DATA with Marker

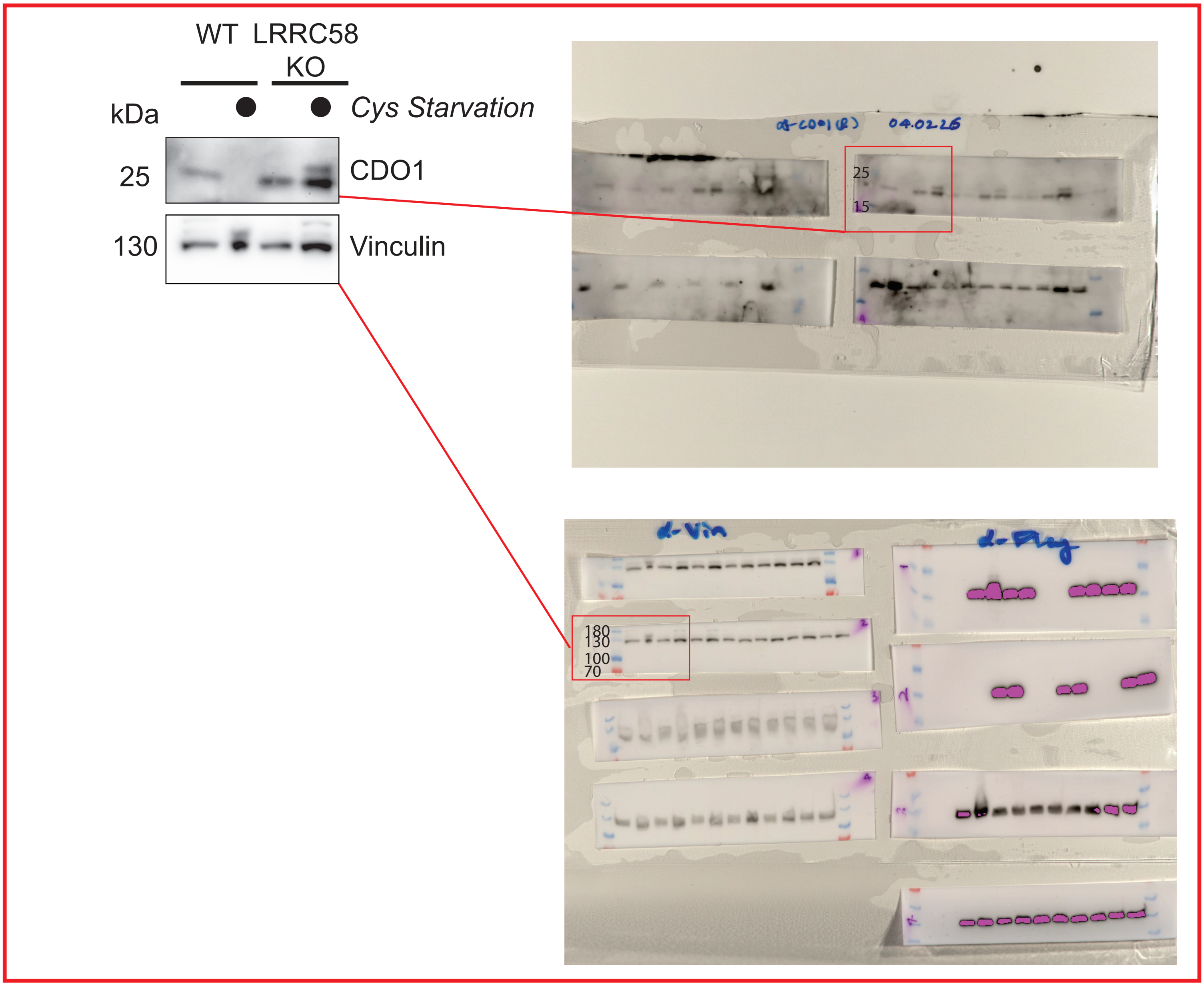

Supplement: Supplementary file 7 — Source Data [file 41467_2026_72524_MOESM7_ESM.zip › SourceData/SourceData_Blot_Fig2f.pdf]
